# What is the impact of antidepressant side effects on medication adherence among adult patients diagnosed with depressive disorder: A systematic review

**DOI:** 10.1177/02698811231224171

**Published:** 2024-02-12

**Authors:** Eleni Niarchou, LH Roberts, Bernard D. Naughton

**Affiliations:** 1 School of Pharmacy and Pharmaceutical Sciences, Trinity College Dublin, Dublin, Ireland; 2 Department of Psychology, University of Buckingham, Buckingham, UK

**Keywords:** Medication adherence, adverse drug reactions, major depressive disorder

## Abstract

**Background:**

Medication adherence is a prerequisite to achieving beneficial treatment outcomes. In major depressive disorder, many patients fail to complete medication regimens, raising concern for poor treatment outcomes. It is usual to experience adverse drug reactions (ADRs) while taking antidepressants, and relative discomfort is reported by patients.

**Aims:**

The present review focuses on the presence of antidepressant-related side effects and the subsequent relationship with medication non-adherence.

**Methods:**

The Preferred Reporting Items for Systematic Reviews and Meta-Analyses (PRISMA) guidelines were followed. Following the preliminary research, the research question and eligibility criteria were created based on the PICO framework. All articles retrieved from the selected databases were exported to Covidence, a Systematic Review managing software tool. Two reviewers assessed the papers to identify the risk of bias using the Joanna Briggs Institute critical appraisal tool for cross-sectional studies. Seven studies with a low–moderate risk of bias fulfilled the eligibility criteria and were conducted from 2013 to 2020 in Europe, Africa and Asia.

**Results:**

The results demonstrated high levels of suboptimal adherence ranging from 46% to 83% amongst the studied population. A variety of side effects were reported by a significant number of participants predominantly with moderate severity. A correlation between the presence of ADRs and suboptimal rates of adherence to antidepressants was found. Somnolence and headaches among other unspecified ADRs were found to increase the dropout rates for selective serotonin reuptake inhibitors.

**Conclusions:**

The present study elucidates the need for effective interventions to facilitate antidepressant adherence and enhance doctor–patient communication, benefiting both the individuals and the healthcare system and leading to better clinical outcomes and reduction of relapse-related costs.

## Introduction

Adherence is a core component of medical treatment and can be defined as compliance with the guidelines provided by the physician regarding the taking of medicines (Keyloun et al., 2017). Medication adherence (MA) has been proven to be a prerequisite to achieving beneficial treatment outcomes, and failure to comply with medication regimes has been associated with negative consequences, not only on an individual level by jeopardising patients’ health but also on the wider community level, amplifying the healthcare costs caused by disease reoccurrence (Marrero et al., 2020).

Research has demonstrated suboptimal levels of adherence to medication in various chronic conditions such as hypertension (Tedla and Bautista, 2016) and HIV (Zhang et al., 2016) as well as in several mental health conditions such as schizophrenia (DiBonaventura et al., 2012) and depression (Ho et al., 2017). Specifically, in major depressive disorder (MDD), the dropout rates (rates of patients failing to complete their medication regimens) fluctuate from 10% to 60%, raising concern for poor treatment outcomes and subsequent increase in hospitalisation and emergency visits rates (Krivoy et al., 2016).

MDD as a disabling condition puts a significant burden on public health systems worldwide, affecting patients’ quality of life by aggravating morbidity and mortality levels, and increasing the need for healthcare services (Ho et al., 2017). Several studies suggest that MDD prevalence rates remain stable with societal and demographic factors influencing fluctuations in different geographical locations (Murphy et al., 2000; Rait et al., 2009; Richter et al., 2019; Schürmann and Margraf, 2018). Yet, others argue that it is expected to be one of the leading causes of illness by the end of the decade (Mathers and Loncar, 2006). MDD symptoms have been proven to decrease with the pharmacological treatment of antidepressant medication, resulting in better health outcomes and decreased mortality (Keyloun et al., 2017).

Antidepressants (ADs) are a core part of the treatment procedure for moderate or severe depression based on the NHS guidelines (NHS, 2021a), the National Institute for Health and Care Excellence (NICE, 2022) and are further corroborated by the literature (Cleare et al., 2015; Kennedy et al., 2016). The pharmacological treatment with antidepressants can be divided into two phases: the initiation/acute phase (6–12 weeks) and the continuation phase (4–9 months) (Keyloun et al., 2017). Several types of antidepressants can be clustered in groups: Selective serotonin reuptake inhibitors (SSRIs) such as fluoxetine, serotonin-noradrenaline reuptake inhibitors (SNRIs) such as venlafaxine, noradrenaline and specific serotonergic antidepressants such as mirtazapine, tricyclic antidepressants (TCAs) such as amitriptyline, serotonin antagonists and reuptake inhibitors such as trazodone and monoamine oxidase inhibitors such as phenelzine (NHS, 2021a).

Previous research has highlighted that adherence to antidepressant medication is often lower than in medication for other chronic conditions such as diabetes or hypertension (Keyloun et al., 2017). Therefore, it is vital to explore the factors that lead to optimal adherence to antidepressants. Factors influencing MA have been associated with characteristics related to the patient, the medication, the illness and the clinical setting (Hassan et al., 2016). In more detail, patient-related factors include beliefs and fears about medicines, gender, patients’ literacy, economic status, social support, etc. Medication-related factors include adverse side effects and desired efficacy, while illness-related factors include the severity of depression and existing comorbidities. Finally, clinical setting factors include follow-up rates and doctor–patient communication (Hassan et al., 2016; Hung, 2014).

During the first period of taking antidepressants, it is usual to experience various adverse drug reactions (ADRs) which differ based on the type of medication prescribed. It is advised to continue the pharmacological treatment despite the ADRs because the benefits outweigh the discomfort caused by the side effects several weeks after the medication initiation. Specifically, SSRIs and SNRIs are usually responsible for agitation or anxiety, digestion, sexual and sleeping disorders, dizziness and headaches. TCAs can cause among others dry mouth, constipation, drowsiness and weight gain (NHS, 2021b).

The literature demonstrates that antidepressant-related side effects persist for more than half of the patients even after taking antidepressants for 75–105 days; this is often underestimated by healthcare providers resulting in poor patient–doctor communication about the prescription (Kelly et al., 2008). Also, treatment dropout due to antidepressant-related side effects is estimated to occur after 6.5–7 weeks, while other reasons result in later discontinuation (Kelly et al., 2008).

### Aim and objectives

This review will focus on the presence of antidepressant-related side effects and the subsequent medication non-adherence as there is no systematic review exploring the effect of the antidepressants’ side effects on adherence. The first objective is to explore the prevalence of ADRs and MA among patients with depression over the last 10 years (2012–2022). Secondly, the authors aim to explore whether the presence of ADRs is associated with lower MA among patients with depression.

## Methods

### Protocol

The Preferred Reporting Items for Systematic Reviews and Meta-Analyses (PRISMA) guidelines (Page et al., 2021) were followed and a study protocol was developed including the search strategy, the eligibility criteria, the data extraction items and the risk of bias assessment.

### Search strategy

Preliminary research was conducted to explore the validity of the research idea. The EBSCO Discovery was used, and more broad search terms were selected. Google Scholar database was also used to investigate the systematic reviews that had already been conducted on this research area. Following the preliminary research, a more comprehensive list of search terms including three search clusters was developed (see Table 1) and the final research question was formulated based on the population, intervention, comparison, outcomes (PICO) framework (Methley et al., 2014) (see Appendix 1, Table A1). A systematic review search was performed on ‘APA PsycArticles’, ‘APA PsycInfo’, ‘MEDLINE’ and ‘Academic Search Complete’. Only research articles that were published in the English language in the last 10 years (2012–2022) in peer-reviewed journals were included by the filters selected in the search procedure. This time period was chosen as the purpose of the study was to explore contemporary data and because trends of antidepressant prescriptions by class are shifting through the decades as shown by Bogowicz et al. (2021).

**Table 1.** Search strategy and keywords.

Final search*	
Search terms	Number of results
Medication adherence	68,100
OR Medication non-adherence	
OR Medication compliance	
OR Medication non-compliance	
Antidepressants	215,548
OR Antidepressant medication	
OR SSRI OR SSRIs	
OR Selective serotonin reuptake inhibitors	
Side effects	726,969
OR Adverse drug reaction	
OR Adverse drug events	
OR Adverse drug effects	
S1 AND S2 AND S3	371
Apply filters	124(after duplicates removed in EBSCO)
English language, peer-reviewed, 2012–2022	

* Conducted on APA PsycArticles, APA PsycInfo, MEDLINE and Academic Search Complete.

### Eligibility criteria

The eligibility criteria were created based on the PICO framework. A primary diagnosis of depression and pharmacological treatment with antidepressants were the inclusion criteria. Animal studies, review articles, conference abstracts and editorials as well as studies with a qualitative design were excluded. Also, studies that focused on specific antidepressant medications or specific antidepressant side effects (e.g. sexual dysfunction) were excluded. This is because the present review aims to focus on multiple and not singular antidepressants and side effects. The focus on specific side effects among the studies would also affect the comparability of the findings. Longitudinal studies were also excluded as the measurements from different timepoints could be impacted by changes in the dose or type of medication. Similarly, studies with significant comorbidities and subsequent polypharmacy were excluded (e.g. HIV or cancer patients). Additionally, studies that focused on a specific population group (children, elderly, patients with another medical diagnosis, apart from depression, etc.) were excluded because of the complexity the management of their medication regimens might present (family members and caregivers could be involved).

### Data extraction

**Figure 1.**
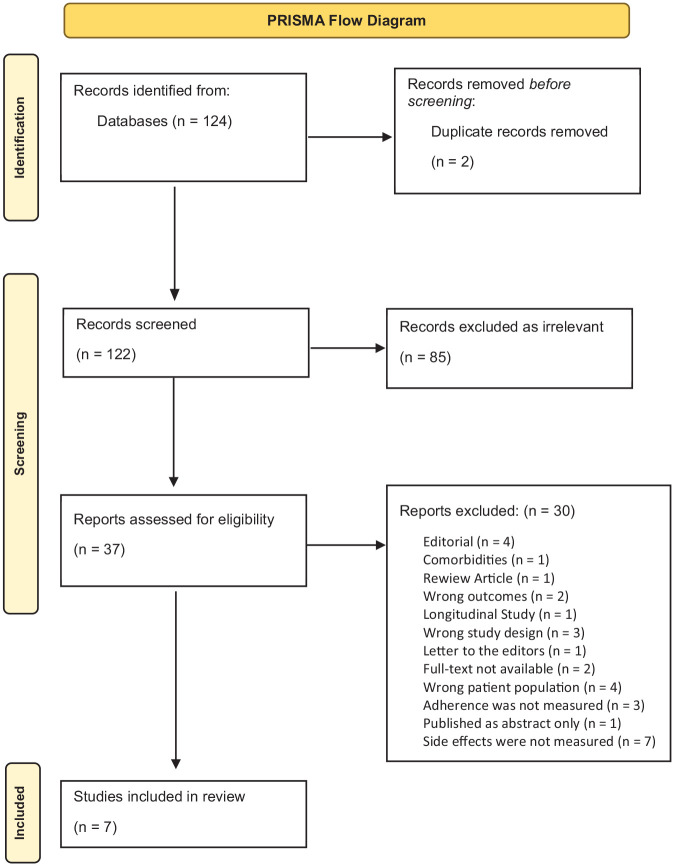
PRISMA flow diagram.

All the articles retrieved from the selected databases after the three search clusters were combined with ‘AND’ and the filters were applied, were exported to Covidence, a Systematic Review managing software (Veritas Health Innovation, Melbourne, Australia). The data extraction was conducted by two reviewers. After the duplicates were removed, the reviewers did an initial screening of the title and abstract of the articles and subsequently reviewed the full text of the studies that were not excluded as irrelevant in the first stage. Conflicts were resolved through discussion meetings amongst the two reviewers until a consensus was reached. There were no instances where consensus was not reached. The screening and selection processes are described in Figure 1.

### Data items

The data extracted were as follows: authors’ name, year of publication, number of participants and their primary diagnosis, country and setting where the study took place, study design, types of antidepressant medication, presence and severity of ADRs, MA levels and clinical outcomes. The limitations of the studies were also examined.

### Study risk and bias assessment

Two reviewers assessed the papers to identify the risk of bias using the Joanna Briggs Institute (JBI) critical appraisal tool for cross-sectional studies (Moola et al., 2020). A high risk of bias is identified for the studies that had a positive answer in less than half (<4) of the criteria, while a low risk of bias is present when the studies scored positive answers in more than 70% (>5.6) of the criteria. The rest of the studies presented a moderate risk of bias according to Moola et al. (2020) as low, medium and high risk of bias classification.

## Results

### Study selection

The study selection process is presented in Figure 1. The final search resulted in 124 studies of which 2 were duplicates and were removed before the screening. In all, 122 papers were included in the first screening stage and 85 of them were clearly irrelevant to the research question and were excluded based on the title and abstract. In all, 37 studies were included in the full-text screening stage. Finally, seven studies fulfilled the eligibility criteria. Three of the selected studies explored the factors associated with antidepressant medication non-adherence, finding ADRs to be one of them, while the other four specifically explored antidepressant side effects and MA.

### Study characteristics

The selected studies were conducted from 2013 to 2020 in Europe (Spain, Germany and France), Africa (Ethiopia) and Asia (Turkey, Nepal and Hong Kong). The timeframe of the studies was from 3 months to 3 years and data were collected at a single point in time for each participant (no longitudinal studies were included). The participants of the final papers were adults over 18 years of age who had a primary psychiatric diagnosis of depression, with the female gender being overrepresented. The number of participants fluctuated from 53 to 50,824 (see Table 2).

**Table 2.** Summary of included studies’ characteristics.

First author and date	Country	Study design	Setting	Participants				Study timeframe
				*N*	Age (years)	Gender (male %)	Primary diagnosis	
Abegaz et al. (2017)	Ethiopia	Prospective cross-sectional study	Gondar University Hospital	217	30.94 ± 8.85	56.2%	MDD	September 2016–January 2017
Marasine et al. (2020)	Nepal	Prospective cross-sectional study	Outpatient Department of B.G. Hospital	174	65 > *x* ⩾ 18	44.25%	Depression	August–October 2019
Kostev et al. (2014)	Germany	Cross-sectional study	1192 General Practices	50,824	>18	32.5%	MDD	January 2009–December 2012
Yau et al. (2014)	Hong Kong	Retrospective cohort study	Prince of Wales Hospital	189	20–88	28.6%	MDD	January 2006–December 2007
De las Cuevas et al. (2014)	Canary Islands, Spain	Cohort study	2 Community Mental Health Centres	145	>30	24%	Mood disorders	September 2011–May 2012
Baeza-Velasco et al. (2019)	France	Cross-sectional study	Department of Emergency Psychiatry and Acute Care of the University Hospital of Montpellier	360	18–77	35.3%	Major depressive episode	May 2012–July 2015
Deniz et al. (2016)	Turkey	Cross-sectional study	Haydarpasa Numune Training and Research Hospital Psychiatry Department	53	33.25 ± 11.29	13.2%	MDD	December 2012 –May 2013

NM: not mentioned.

### Methodological evaluation

Five of the selected studies had a cross-sectional design while the other two had a retrospective approach to collecting the data. Only one study was conducted in general practices and one in community-based mental health centres. Participants for the rest of the studies were hospital psychiatric patients. The clinical diagnosis of depression was one of the inclusion criteria and was determined by the psychiatrists based on the International Classification of Diseases (ICD-10) and the Diagnostic and Statistical Manual of Mental Disorders 4th Edition (DSM-IV). Regarding the ADRs and the MA, the studies that adopted a cross-sectional design utilised self-reported questionnaires to collect the data. The retrospective design studies used clinical information reported by physicians from computerised databases. Table 3 describes in detail the questionnaires used for each outcome variable. It can be noted that the majority of the studies utilised similar psychometric tools for data collection.

**Table 3.** Summary of data collection process.

First author and date	Country	Outcome variables	Instruments used			Other instruments	Data collection route
			ADR	MA	Clinical outcomes		
Abegaz et al. (2017)	Ethiopia	(a) Level of adherence to antidepressants (b) Patient clinical outcomes (c) ADRs	Naranjo ADR probability scale & The Antidepressant Side-effect Checklist (ASEC)	Morisky Medication Adherence Measurement Scale-Eight (MMAS-8)	Patient Health Questionnaire (PHQ-9)		Clinical psychiatrist-pretested structured data collection tool
Marasine et al. (2020)	Nepal	(a) Level of adherence to antidepressants (b) ADRs	Naranjo ADR Probability Scale and ASEC	Morisky Green Levine Adherence			Data were collected prospectively from patients and prescriptions by communicating with psychiatrists
Kostev et al. (2014)	Germany	(a) ADRs (b) Impact of ADRs on dropout rate	Retrospective design				Disease Analyzer database (IMS HEALTH)*
Yau et al. (2014)	Hong Kong	(a) Non-continuous antidepressant use (b) Relapse/reoccurrence of depressive episodes	Retrospective design				Data retrieval using CDARS**
De las Cuevas et al. (2014)	Canary Islands, Spain	(a) Predictors of adherence to antidepressant treatment	The ASEC - Global Adverse Reaction Severity Index (GARSI), - Positive Side-Effect Distress Index and - Positive Side-Effect Total	Morisky 4-item Questionnaire and Drug Attitude Inventory (DAI-10)	ICD-10 diagnosis, CGI-Severity of illness scale, CGI-Improvement scale (doctors) and The Beck Depression Inventory II (BDI-II)	The Beliefs about Medicines Questionnaire and The Leeds Attitude Toward Concordance scale (LATCon)	Self-reported brief socio-demographic survey and questionnaires
Baeza-Velasco et al. (2019)	France	(a) Optimal MA rates (b) Predictors of suboptimal adherence	Morisky 4-Items Questionnaire (factor 3)	Morisky 4-Items Questionnaire (factor 1, 2), MARS-10 and DAI-10	The Mini International Neuropsychiatric Interview and BDI-13	Childhood Trauma Questionnaire, Negative Life Experiences Questionnaire and Physical Pain Intensity Rating Scale	Interviews by psychiatrists and self-reported questionnaires
Deniz et al. (2016)	Turkey	(a) CYP2C19 alleles and genotype group (b) ADRs (c) Adherence to antidepressants	Toronto Side Effects Scale	Morisky 4-Items Questionnaire (Turkish validated version)		Blood samples and High Pure PCR Template Preparation Kit for genomic DNA isolation	Clinical interview and blood samples

* Data obtained from the computer systems from the practices of general practitioners and specialists throughout Germany.

** Clinical Data Analysis and Reporting System (CDARS) which stores longitudinal clinical information.

### Risk of bias for included studies

JBI critical appraisal tool for cross-sectional studies (Moola et al., 2020) was used to assess the quality of the studies. All the selected papers scored very high on the items and the risk of bias was low (See Table 4).

**Table 4.** Risk of bias assessment.

JBI criteria	Abegaz et al. (2017)	Marasine et al. (2020)	Kostev et al. (2014)	Yau et al. (2014)	De las Cuevas et al. (2014)	Baeza-Velasco et al. (2019)	Deniz et al. (2016)
1. Clearly defined inclusion/exclusion criteria		√	√	√		√	√
2. A detailed description of the study subjects and the setting	√	√	√	√	√	√	√
3. A reliable way of measuring exposure	√	√	√	√	√	√	√
4. Use of objective, standard criteria for measurement of the condition	√	√	√	√	√	√	√
5. Identification of confounding factors		√		√		√	√
6. Use of strategies to deal with confounding factors		√		√		√	√
7. A reliable and valid way of measuring outcomes	√	√	√	√	√	√	√
8. Use of appropriate statistical analysis	√	√	√	√	√	√	√
Total score	5/8	8/8	6/8	8/8	5/8	8/8	8/8
Risk of bias	Moderate	Low	Low	Low	Moderate	Low	Low

### Results of individual studies

#### Adverse drug reactions

Five studies explored the different types of side effects in detail (Abegaz et al., 2017; De las Cuevas et al., 2014; Deniz et al., 2016; Kostev et al., 2014; Marasine et al., 2020). As Table 5 demonstrates, the most prevalent ADRs were similar to all the studies with slight differences in the hierarchy. Most of the studies had a variety of antidepressant medications being prescribed except for the studies of Kostev et al. in Germany and Deniz et al. in Turkey which explored solely SSRI prescriptions. It should also be noted that the German study did not report the total percentage of participants that encountered side effects. The other four studies reported the percentage of patients that experienced ADRs which was significantly high (over 50%). Additionally, the three studies that explored the severity of the ADRs reported a high prevalence of moderate severity side effects followed by severe ones (Abegaz et al., 2017; Marasine et al., 2020). Appetite dysregulation (weight gain, loss of appetite), sleep disorders (insomnia, drowsiness), dry mouth and headaches were some of the most common side effects. Drowsiness and dry mouth were found to be the ‘most troubling’ in the Turkish study by Deniz et al.

**Table 5.** Summary of antidepressant medication and related side effects.

First author and date	Country	Antidepressant medication	ADR	Medication adherence (non-adherence %)
Abegaz et al. (2017)	Ethiopia	Amitriptyline Fluoxetine Miscellaneous Imipramine	Weight gain Loss of appetite Dry mouth Drowsiness Constipation Insomnia	57.1%
Marasine et al. (2020)	Nepal	SSRIs: fluoxetine, sertraline, escitalopram SNRIs: duloxetine, venlafaxine TCA: amitriptyline Atypical antidepressants: bupropion, mirtazapine	Insomnia Anxiety Dry mouth Weight gain Loss of appetite Agitation	52.3%
Kostev et al. (2014)	Germany	SSRIs	Symptoms related to the digestive system Sleep disorders Heart rhythm disorders Dizziness Malaise and fatigue Headache	–
Yau et al. (2014)	Hong Kong	Mirtazapine, SSRI, TCA and its related antidepressant, SNRI, Others	–	46%
De las Cuevas et al. (2014)	Canary Islands	Antidepressants, TCAs, SSRIs, SNRIs and benzodiazepines, antipsychotics conventional, atypical mood stabilisers	Dry mouth Insomnia Weight gain Headache Feeling lightheaded upon standing	46.2%
Baeza-Velasco et al. (2019)	France	Antidepressants, anxiolytics, antipsychotics, hypnotics, mood stabilisers, other drugs	–	70.3%
Deniz et al. (2016)	Turkey	SSRIs (citalopram, escitalopram or sertraline)	Drowsiness Dry mouth Fatigue Sweating Decreased appetite Agitation Decreased libido Weight loss (and more)	83%

#### MA and the impact of antidepressant side effects

The results of the selected studies demonstrate that the levels of antidepressant non-adherence or low adherence among patients with depression are very high fluctuating from 46% to about 83% (see Table 5). Side effects while taking antidepressants appeared to be associated with the medication non-adherence levels in all studies, except for the study conducted in Ethiopia by Abegaz et al. (2017) where they explored these variables independently suggesting that future research should aim to establish their correlation. More specifically, three of the selected studies demonstrated that the presence of side effects increases the odds of the patients being less adherent to their antidepressant medication regimes (Kostev et al., 2014; Marasine et al., 2020; Yau et al., 2014). Kostev et al. (2014) also highlighted the impact that specific side effects have on the adherence level, with somnolence increasing the odds of the dropout rate 8.42 times. It should be noted that in this paper non-adherence was determined based on dropout rates and other indices of adherence (e.g. missed doses) were not reported. Moreover, in the study conducted in France in 2018 by Baeza-Velasco et al., patients with suboptimal levels of adherence had higher scores in the ‘Negative medication side effects’ questionnaire, yet the causal association was not found to be significant in the logistic regression model (Baeza-Velasco et al., 2019). Similarly, in the study conducted by Deniz et al., in 2016, more adherent participants were found to have lower scores in the frequency, severity and intensity of side effects; however, this was not found statistically significant. De las Cuevas et al. (2014) also found non-adherent patients to score higher in the ASEC-GARSI questionnaire measuring the number and severity level of the ADRs. However, a causal effect was not specifically explored (see Table 6).

**Table 6.** Summary of statistical results.

First author and date	Country	ADRs and MA	
		Odds ratio (OR)	Other outcome
Abegaz et al. (2017)	Ethiopia	–	
Marasine et al. (2020)	Nepal	1.173 (0.42–3.25), *p* < 0.001	
Kostev et al. (2014)	Germany	Somnolence: 8.42 (1.86–38.10), *p* = 0.0056 Headache: 1.32 (1.05–1.66), *p* = 0.0164 Unspecified ADR: 1.61 (1.21–2.15), *p* = 0.0012	
Yau et al. (2014)	Hong Kong	2.27 (1.12–4.63), *p* = 0.024	
De las Cuevas et al. (2014)	Canary Islands, Spain	–	Non-adherent patients scored higher on the GARSI scale regarding the number and severity of ADRs ASEC-GARSI (*M* (SD)): adherent: 0.71 (0.41), non-adherent: 0.87 (0.48), *p* = 0.035
Baeza-Velasco et al. (2019)	France	Not significant	Patients with suboptimal adherence had higher levels of negative antidepressant side effects (1.1 ± 0.8 vs 0.9 ± 0.8; *t* = −2.03; *p* = 0.046) but this was not significant in the logistic regression model
Deniz et al. (2016)	Turkey	Not significant	Lower frequency, severity and intensity of mean side effects resulted in higher adherence, but the difference between these groups was not found to be statistically significant (*p* > 0.05)

## Discussion

### Medication adherence

One of the primary aims of this systematic review was to explore the levels of MA among patients diagnosed with depressive disorder. The results demonstrated high levels of non-adherence with most studies reporting that about one-half of the participants did not adhere to the medication regimes. This corroborates previous findings, suggesting that MA in antidepressants is a challenging situation that requires attention and effective interventions to reduce the risks embedded in the significant dropout rates (Ho et al., 2017; Marrero et al., 2020). It is well established that low levels of MA are linked with poor clinical outcomes and higher relapse in depressive disorder (Ho et al., 2017); therefore, this finding should not be overlooked. The selected studies’ samples suggest a diverse cultural and social background having been conducted in a variety of countries and continents, elucidating that despite the healthcare system differences, this is a shared challenge that needs to be faced effectively. However, some studies have illustrated cultural differences in the levels of compliance with antidepressants, showcasing that several population groups such as Latinos, African Americans and immigrants demonstrate lower adherence levels (Hung, 2014; Interian et al., 2013). The present study could not capture such differences and it is suggested that future systematic reviews should explore the influence of various racial backgrounds comprehensively to reinforce inclusive interventions that respect individual differences. Moreover, it is important to highlight that the selected studies were conducted in both outpatient and inpatient settings. Research has shown that the levels of adherence to outpatient treatment visits differ between patients that are hospitalised and patients in outpatient units. Specifically, it was found that hospitalised patients rarely attend such visits, even though they usually have a longer medical history of depression and higher severity of illness (Karpov et al., 2018). It would be interesting to explore if such differences apply to MA levels between these patient groups.

### Adverse drug reactions

This study aimed to investigate the prevalence of antidepressant-related side effects. There was adequate homogeneity regarding the types of antidepressants that were prescribed in the selected studies, with most of the studies focusing on multiple pharmacological treatment options. The drawback of this broader methodological approach is that it is not possible to conclude about the different types of antidepressants and their specific side effects. However, the findings showed a variety of side effects. In the majority of the studies, a considerable number of participants reported experience of ADRs, usually of moderate severity. These findings are independently important because patients experiencing ADRs usually receive prescriptions for lower doses than the optimal titration dose, resulting in longer periods of treatment and prolonging of the disease. It is also understandable that even if the side effects do not result in dropping out of treatment, they jeopardise the patient’s quality of life (Kelly et al., 2008). Additionally, it is known that patients’ beliefs and fears about antidepressant medication influence the experience of side effects (Ho et al., 2017) but it can also be argued that the experience of ADRs can impact the perceptions about antidepressants, especially when side effects related to sexual dysfunction are experienced (Clayton et al., 2014). Therefore, it is important to acknowledge the subsequent negative psychological effects that several ADRs can have on patients who are already severely impacted by depression.

### The effect of ADRs on MA

The findings among all selected studies confirmed the correlation between the presence of ADRs and suboptimal rates of adherence to antidepressant medication. Almost all studies that tested a potential causal effect using regression models found that ADRs increase the odds of patients presenting lower adherence levels. Only one study explored the relation of specific side effects with the levels of MA, highlighting that somnolence and headaches increase the dropout rates for SSRI treatment regimens (Kostev et al., 2014). This is supported by the findings of another study that explored the severity and intensity of the reported side effects, where somnolence was found to be amongst the most troubling (Deniz et al., 2016). These findings are of high importance because they highlight the need to develop interventions that target such side effects to mitigate the negative impact of non-adherence. Healthcare practitioners could provide educational information to patients before prescription in regard to the potential side effects that they might encounter and their characteristics (acute or persistent) (Kelly et al., 2008). In research conducted on the Chinese population about adherence to ART treatment for HIV, it was found that there is a mediating effect of self-efficacy in the impact that ADRs have on MA (Zhang et al., 2016). Patients with higher adherence self-efficacy were better at dealing with discomfort related to side effects and were more likely to follow the physician’s directions on taking the medication. It is demonstrated in Bandura’s Social Cognitive Theory (1998) that self-efficacy can enhance motivation and influence the effect and behaviour of individuals resulting in better health behaviours (Zhang et al., 2016). Future research should explore the potential mediation effect of self-efficacy on antidepressant medication adherence and the presence of ADRs. This could lead to the development of successful interventions that would facilitate the regulation of the discomfort caused by ADRs and reduce the dropout rates.

### Limitations and modifications

This systematic review has several limitations. Firstly, grey literature (unpublished research articles) was not included in the search strategy resulting in a potential publication bias. Also, only English language articles were included limiting the breadth of the represented populations. However, studies from almost all continents were included. Secondly, the search strategy was limited to a selection of specific databases that the EBSCO database provided access to, although this did include Medline. Thirdly, the heterogeneity of the methodological approach and statistical analysis used in the selected studies should be taken into account in conjunction with the variety of instruments used to measure the outcome variables. Future systematic review studies could expand their searches in more databases and decide upon specific psychometric instruments in their review protocol. Furthermore, it is suggested that more efficient methods of monitoring side effects should be developed to account for polypharmacy and multimorbidity which is the reality of clinical practice.

## Conclusion

The level of optimal MA is often overestimated by physicians, while the level of side effects experienced by the patients is underestimated (Kelly et al., 2008). Therefore, the present systematic review is of significant importance to the patients, elucidating the urgent need to develop effective interventions to facilitate adherence to antidepressants and enhance the communication between the patients and the doctors. This can benefit both the individuals and the healthcare system leading to better clinical outcomes and subsequent reduction of relapse-related costs.

## Appendix 1

**Table A1.** PICO framework.

P	I	C	O
Patient, population or problem	Intervention or exposure	Comparison	Outcome(s)
Patients diagnosed with depressive disorder	Receiving antidepressant medication	Existence and severity of side effects	Levels of medication adherence
